# Exposure to Lead, Cadmium, Mercury and Arsenic Among Asian and Non-Asian Children and Adolescents in the United States: NHANES 2015–2018

**DOI:** 10.1007/s10903-024-01634-1

**Published:** 2025-01-03

**Authors:** Lanxin Song, Ondine S. von Ehrenstein

**Affiliations:** 1 Department of Epidemiology, Fielding School of Public Health, University of California, Los Angeles, PO Box 951772, Los Angeles, CA 90095-1772 USA; 2 Department of Community Health Sciences, Fielding School of Public Health, University of California, Los Angeles, PO Box 951772, Los Angeles, CA 90095-1772 USA

**Keywords:** NHANES, Heavy metals, Asian, Children, Diet, Mediation

## Abstract

Higher concentrations of heavy metals were reported mainly among adult Asian persons compared to other racial/ethnic groups in earlier NHANES cycles’ studies. We aimed to examine concentrations of metals among Asian children/adolescents compared to children/adolescents identifying with other racial/ethnic groups, considering socio-demographic factors and potential mediation by fish/shellfish consumption. Using NHANES data (2015–2018), 5293 participants (1–19 years) with blood/urinary measurements of lead, cadmium, mercury and arsenic were included. Survey-weighted adjusted generalized linear models assessed differences in log-transformed metal concentrations between Asian and non-Asian participants, considering potential effect measure modification by parental education. Causal mediation effects of recent fish/shellfish consumption were estimated. Log-transformed metal concentrations were higher among Asian than among non-Asian children/adolescents. Lower parental education was associated with higher concentrations of cadmium and methylmercury among Asian participants, and effect measure modification was suggested. Proportions of the mercury exposure disparity mediated by recent fish/shellfish consumption were 9.1% (95% CI 2.6, 17.3%) for fish, and 5.7% (0.5, 12.0%) for shellfish, with similar findings for methylmercury. Overall, Asian identification was associated with higher toxic metal exposure, and among Asian children/adolescents those with parents with lower education were particularly affected. Dietary fish/shellfish intake explained a portion of the disparities. Prevention efforts should identify sources of elevated metal exposure focusing children and adolescents while considering diverse backgrounds and dietary habits including high fish consumption.

**Supplementary Information:**

The online version contains supplementary material available at 10.1007/s10903-024-01634-1.

## Background

Child and adolescent exposure to heavy metals has been shown to increase risks for a range of adverse health outcomes [1], including impairment of the nervous system, neurological and behavioral problems, certain cancers, lung disease, renal disease, immune suppression, and cardio-metabolic disorders. While several demographic and socio-economic factors have been found to be predictors of metal exposure in the U.S. population [2–6], studies addressing these factors focusing children and adolescents among Asian population groups in the U.S., are scarce.

Based on earlier National Health and Nutrition Examination Survey (NHANES) data [7], Asian participants aged 1 year through adulthood had higher concentrations of several toxic metals compared to other racial/ethnic groups, corroborating previous research [2, 5, 8–11]. Higher urinary concentrations of arsenic, cadmium, mercury and lead among Asian participants were also observed in the Study of Women’s Health Across the Nation and a few smaller studies in Canada and UK [12–15]. Nationwide biomonitoring data and large cohort studies in Asian countries reported more elevated blood and urinary metal concentrations compared to studies among participants of Asian-origin in the U.S [16–18].

Among adults, concentrations of several heavy metals have been found to differ by socioeconomic status (SES) indicators, such as income-to-poverty ratio, educational status, and neighborhood deprivation, with the lower SES groups often experiencing higher exposures [4, 5, 8, 19–21]. Among Asian groups specifically, in an earlier NHANES study, inverse associations were observed between SES and cadmium, as well as between education level and lead, not considering children separately [8].

Among children, a study in California reported elevated urinary lead levels linked with neighborhood deprivation only among Asian girls [20]. A European study, suggested childhood lead exposure to be higher in lower-SES groups while mercury and arsenic exposure were higher in higher-SES groups, regardless of race/ethnicity [19]. Similarly, in an Indian study, blood lead in children was negatively associated with maternal education and family income [22]. Conversely, higher blood lead was reported for younger children whose fathers had a higher education level in Poland [23]. Thus, findings are mixed and data are scarce focusing on SES in relation to toxic metal exposure among U.S. Asian children and adolescents.

Dietary intake, such as seafood, rice and alcohol, has been suggested as an important exposure source for arsenic and mercury, especially for population groups who consume these foods more frequently like both, Asian children [9, 24, 25] and adults [2, 9, 10, 25, 26]. Estimated dietary intake of arsenic and mercury was primarily attributed to fish consumption among Asian populations, which was markedly associated with urinary arsenic and blood mercury compounds’ concentrations [9, 27, 28]. Seafood consumption was also identified as predictor of blood mercury concentration, especially among Asian women of reproductive age [10]. While rice was suggested to be an important source of arsenic exposure for Asian children [9, 24, 25], other dietary factors have been rarely considered among this age group.

### Conceptual Framework

Our analysis is driven by the theory of Fundamental Causes that depicts key resources connected to SES such as knowledge, money, prestige, and beneficial social connections are determinants of health-related outcomes and behaviors [29]. This is extended by the theory of environmental justice addressing the non-equitable distribution of environmental risks and benefits [30]. Additionally, we rely on self-reported racial/ethnic identification as a concept of race and racism based on the growing consensus that racial/ethnic health inequities result from social, economic and political determinants of health [31].

Our objective was therefore to examine potential disparities in concentrations of lead, cadmium, methylmercury and arsenic in urinary and blood specimens comparing Asian vs. non-Asian participants aged 1–19 years using NHANES data (cycles 2015–2016, 2017–2018), considering the role of parental education and other SES related factors as potential effect measure modifiers. We further sought to examine whether recent fish and shellfish consumption mediated any potential exposure disparities.

## Methods

### Data Source

The NHANES includes a series of cross-sectional surveys to produce nationally representative estimates of the U.S. population beginning 1999 annually, released in 2-year cycles. The cycles of 2015–2016/2017–2018 slightly differed from previous cycles in sampling design and are the most recent cycles conducted prior to the Covid-19 pandemic. Detailed study designs and estimation procedures are described elsewhere [32]. NHANES collects demographic, health, and nutrition data through interviews and questionnaires, complemented by standardized physical examinations and the collection of biological specimens (blood/urine) for laboratory analysis in a mobile examination center (MEC). We combined data from the 2015 to 2018 cycles for our analysis.

### Study Population

The study encompasses 7184 participants aged 1–19 years, including both genders and grouped into six racial/ethnic categories (non-Hispanic White, non-Hispanic Black, Mexican, Other Hispanic, other non-Hispanic and Asian), with an oversampling of underrepresented subgroups, including Asian Americans [33]. We excluded 1891 participants missing metal measurements resulting in 5293 participants in the final analytic sample (Fig. S1). Data from the household reference person, typically a parent, provided parental educational information [34]. The NHANES protocol received NCHS Research Ethics Review Board Approval, and is in compliance with the Department of Health and Human Services Policy for the Protection of Human Research Subjects (further details at https://www.cdc.gov/nchs/nhanes/irba98.htm) [35]. All respondents gave informed written consent. Parents or guardians granted written permission for those under 18, and children aged 12–17 provided assent [36, 37]. Data are publicly accessible at https://wwwn.cdc.gov/nchs/nhanes/Default.aspx.

### Exposure Assessment

Metals of interest in this study were selected a priori and include lead, cadmium, mercury and mercury species in whole blood samples as well as total arsenic and arsenic species in urine samples [34]. We retained metals with measurements > 70% at or above the limit of detection (LOD) and treated below–LOD values as per CDC recommendations (LOD/√2). Metals retained are blood lead (µg/dL), blood cadmium (µg/L), blood mercury (µg/L), blood methylmercury (µg/L), and in urine, total urinary arsenic (µg/L), arsenous acid (As(OH)3) (µg/L), monomethylarsonic acid (MMA) (µg/L) and dimethylarsinic acid (DMA) (µg/L).

### Socioeconomic, Demographic and Fish Consumption Information

Demographic/socioeconomic information included age (year), sex/gender, height, weight, U.S.-born (yes/no), parental education (< high school; high school–some college; college+), family income levels (< $20,000; $20,000 to < 45,000; $45,000 to < 75,000; $75,000+), and family size (1–7 family members) [7]. Fish/shellfish consumption was assessed as binary variables indicating whether or not a participant had consumed fish/shellfish in the past 30 days.

### Statistical Methods

Box plots displayed metal concentration distributions by race/ethnicity and socio-demographic factors. We followed NHANES guidance for subsample weights and data variance to ensure the validity of our findings. Due to right-skewed metal exposure data, we log-transformed values for normal distribution approximation. Survey-weighted generalized linear models were computed for log-transformed metal measurements, adjusting for potential confounders and other covariates selected based on previous literature [2–4, 9, 35], including child age, sex/gender, family income, parental education level, US-born status, BMIz and NHANES survey cycle. Urinary measurements were additionally adjusted for creatinine (mg/dL) to account for variations in urine dilution. The “non-Asian” group, including all White, Black, Mexican and Hispanic participants, was used as reference group.

We explored associations between parental education and the metals in adjusted linear models stratified by Asian and non-Asian group. To further assess possible effect measure modification, interaction terms for Asian vs. non-Asian identification and parental education were added to the main models. In sensitivity analyses, we assessed birthplace and income in the adjusted models stratified by Asian, and non-Asian group. In further sensitivity analysis, we adjusted the main models for family size (as an SES indicator and a proxy of enrolling multiple participants from the same household), removed BMIz from the adjusted models as the latter may be limited by extreme BMI values; and conducted models without creatinine adjustment for urinary measures, and also used “only white” as a reference group.

We assessed fish/shellfish consumption as a potential mediator between Asian identification and higher metal exposure using model-based causal mediation analysis [36]. First, a mediation model assessed the conditional distribution of fish/shellfish consumption given race/ethnicity (Asian vs. non-Asian) and other covariates using adjusted logistic regression. To estimate the total effect (TE) of Asian identification related to metal exposure, the average causal mediation effect (ACME) and proportion mediated (PM) by fish/shellfish consumption [36], a linear “outcome model” regressing metal concentration on Asian vs. non-Asian group, fish/shellfish consumption and other covariates was computed.

All analyses were performed using R version 4.1.1. The “mediation” package and “survey” packages were used for the causal mediation analysis and to accommodate weighted survey data.

## Results

### Descriptive Statistics

Our sample comprised of 460 Asian and 4833 non-Asian children and adolescents (Table 1). Overall, the median age was 9 years with approximately equal proportion of males and females. The Asian group included a slightly larger proportion of males (54% vs. 50%) and had a lower average BMIz compared to non-Asian participants. Participants were predominantly born in the U.S., both Asian and non-Asian. The proportion with parental education “equal or higher than college” was greater among Asian (54%) than among non-Asian participants (19%) and more Asian participants had higher family incomes. Fish (55% vs. 43%) and shellfish (42% vs. 32%) consumption, respectively, was reported more frequently among Asian than among non-Asian participants.

**Table 1:** Characteristics of children and adolescents in NHANES 2015–2018 (n = 5293)

		Overall n = 5293	Asians n = 460	Non-Asians n = 4833
Variable				
Age (years, SD)		8.99 ± 5.22	10.03 ± 5.12	8.89 ± 5.22
Age categories (years)	1–2	640 (12.1)	21 (4.6)	619 (12.8)
	3–4	676 (12.8)	67 (14.6)	609 (12.6)
	5–11	2313 (43.7)	193 (42.0)	2120 (43.9)
	12–19	1664 (31.4)	179 (38.9)	1485 (30.7)
BMI-Z score		0.61 ± 1.18	0.14 ± 1.14	0.66 ± 1.17
Race/ethnicity (n, %)	Mexican	1057 (20.0)	–	1057 (21.9)
	Other Hispanic	545 (10.3)	–	545 (11.3)
	Non-Hispanic White	1574 (29.7)	–	1574 (32.6)
	Black	1189 (22.5)	–	1189 (24.6)
	Asian	460 (8.7)	–	–
	Other Non-Hispanic	468 (8.8)	–	468 (9.7)
Sex (n, %)	Male	2673 (50.5)	250 (54.3)	2423 (50.1)
	Female	2620 (49.5)	210 (45.7)	2410 (49.9)
Country of birth (n, %)	US	4957 (93.7)	340 (73.9)	4617 (95.5)
	Others	336 (6.3)	120 (26.1)	216 (4.5)
Income categories	< 20,000	1033 (20.8)	31 (7.4)	1002 (22.1)
	≥ $20,000 to < $45,000	1536 (31.0)	106 (25.4)	1430 (31.5)
	≥ $45,000 to < $75,000	992 (20.0)	79 (18.9)	913 (20.1)
	75,000+	1394 (28.1)	201 (48.2)	1193 (26.3)
Parental education (n, %)	< High school	1167 (22.7)	53 (12.4)	1114 (23.6)
	High school/some college	2874 (55.8)	143 (33.4)	2731 (57.8)
	≥ College	1111 (21.6)	232 (54.2)	879 (18.6)
Survey cycle	2015–‘16	2600 (49.1)	192 (41.7)	2408 (49.8)
	2017–‘18	2693 (50.9)	268 (58.3)	2425 (50.2)
Fish eaten during past 30 days (n, %)	Yes	1998 (43.9)	197 (55.0)	1801 (42.9)
Shellfish eaten during past 30 days (n, %)	Yes	1495 (32.7)	152 (42.3)	1343 (31.9)

Continuous variables: mean ± SD; Categorical variables: n (%). For parental education, < High School = less than High school graduate/GED; High School/Some College = High school graduate/GED or some college or associates (AA) degree; ≥ College = college graduate or higher. BMI-Z score refers to the standard deviation greater (positive value) or smaller (negative value) than the median of the reference [CDC 2000 growth charts (aged 2–19 years) and WHO growth standards (aged 12–24 months)]; percentages shown based on numbers of non-missing

*NHANES* National Health and Nutrition Examination Survey

**Fig. 1:**
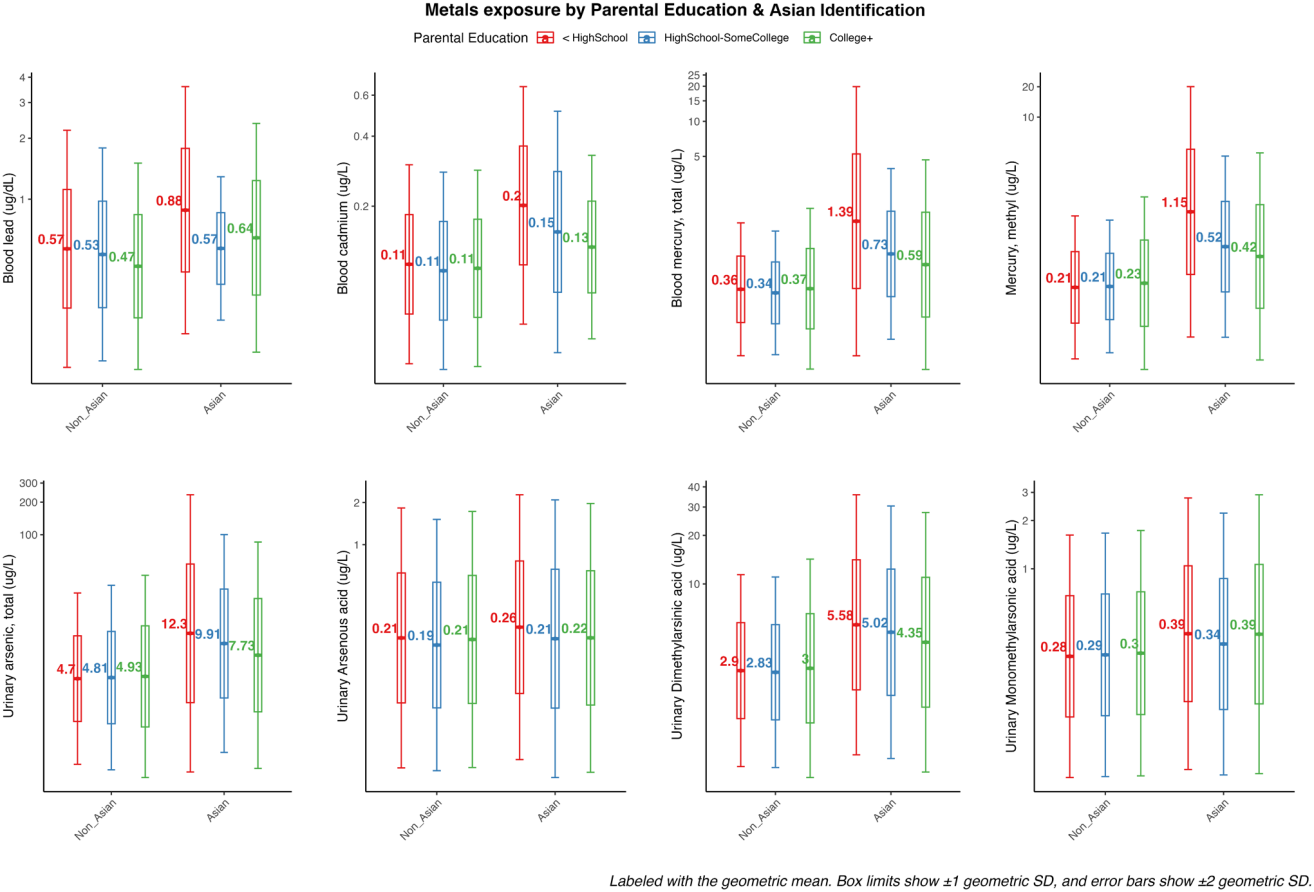
Distribution of metals concentrations by parental education levels for non-Asian and Asian participants aged 1–19 years in NHANES 2015–2018. *Note* NHANES = National Health and Nutrition Examination Survey. Figures are labeled with geometric means, and error bars (± 1 and ± 2 geometric standard deviations)

Asian children and adolescents had on average higher concentrations of all metal compounds compared to the group of non-Asians (Fig. S2) and in comparison with each racial/ethnic subgroup separately (Fig. S3), except for As(OH)3. The differences in metal concentrations among Asian participants varied by metal. Among Asian children and adolescents, those with parents who did not complete high school had higher average metal concentrations compared to those with parents who had higher educational achievements (Fig. 1). In contrast, non-Asian children showed similar cadmium, mercury and arsenic concentrations across different levels of parental education (Fig. 1). For example, blood lead levels ranged from 0 to 5 µg/dL across all groups, while the highest levels were identified among  Asian children/adolescents whose parental education was less than high school, with a geometric mean more than 1 geometric SD higher compared to non-Asian children/adolescents. For mercury and methylmercury, levels ranged from 0 to 8 µg/L, with Asian children/adolescents whose parents had high educational attainment (college+) having geometric means 1 geometric SD higher than non-Asian children/adolescents, and Asian children/adolescents with parents with less than high school having means 2 standard deviations higher than non-Asian children/adolescents.

### Adjusted Results for Asian Identification and SES Measures with Exposure to Metals

**Fig. 2:**
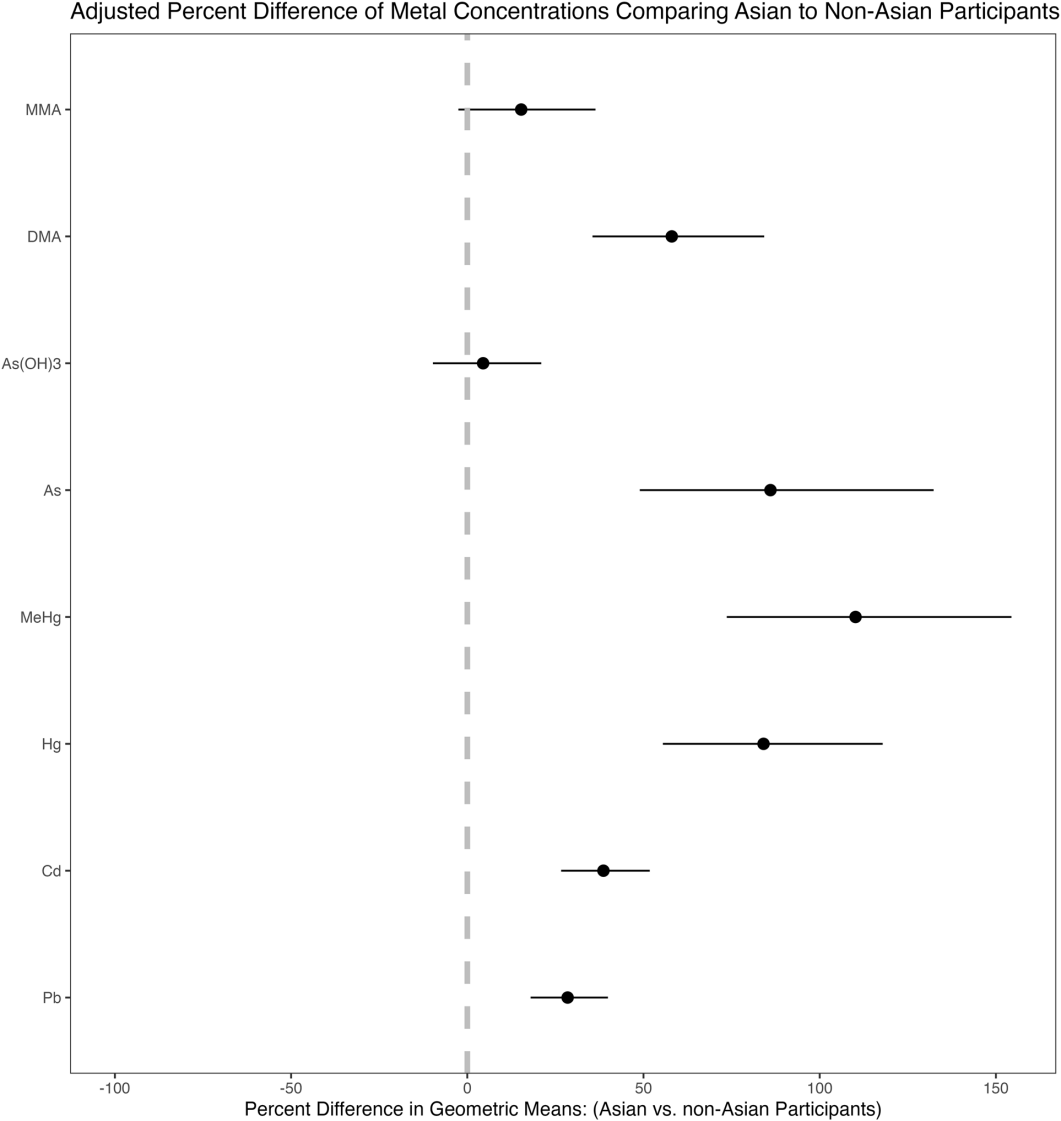
Adjusted percentage difference in metal concentration geometric means among Asian compared to non-Asian children/adolescents in NHANES 2015–2018. *Note* The points represent the percentage difference, and the error bars indicate the 95% confidence intervals. A percentage difference above 0 indicates higher metal concentrations among Asians compared to non-Asians. Metals include lead (Pb), cadmium (Cd), mercury (Hg), methylmercury (MeHg), total arsenic (As), dimethylarsinic acid (DMA), and monomethylarsonic acid (MMA). Models are adjusted for age, sex/gender, family income, US-born, BMI-Z score, cycle and parental education level, with sampling weighting applied; urinary measurements are adjusted for creatinine

After adjusting for age, sex/gender, family income, US-born, BMI-Z score, cycle and parental education level, metal concentrations remained elevated related to Asian identification (vs. non-Asian), except for As(OH)3 (Fig. 2). The adjusted differences in geometric means of metal concentrations between Asian and non-Asian children and adolescents were 29% (95% CI 17%, 41%) for lead; 39% (26%, 53%) for cadmium; 84% (54%, 120%) for mercury; 110% (71%, 158%) for methylmercury; 95% (60%, 137%) for total arsenic; 64% (45%, 86%) for DMA; and 20% (4%, 39%) for MMA (Fig. 2).

Table 2 presents the relative association between Asian identification, parental education, and their interaction on metal exposure, both unadjusted and adjusted for other covariates. When considering the interaction between Asian identification and parental education, statistically significant interaction effects were estimated for cadmium, mercury and methylmercury. For instance, while not adjusting for other covariates, being Asian, compared to non-Asian, was associated with a 71% higher mercury concentration among those with college or higher parental education. The difference in mercury levels between Asian and non-Asian children/adolescents were more pronounced for those whose parents have educational attainment “less than high school” or “high school to some college” compared to those with “college or above”. For parental education alone, without considering Asian identification, no statistically significant association was estimated with mercury levels. Further adjustment for other covariates did not change the direction or magnitude of the associations, while leading to wider confidence intervals due to reduced sample sizes. Computing the adjusted generalized linear regression models stratified by Asian and non-Asian participants indicated for the Asian group that lower parental education related to higher concentrations of lead, cadmium, mercury, methylmercury and total arsenic, however several 95% CI were wide likely due to the limited number in this group. For the non-Asian group overall, lower parental education related to higher lead concentration and lower mercury or methylmercury concentration (Table S1). Using White participants only as reference group in sensitivity analysis, similarly, statistically significant interactions between parental education levels and “Asian” were indicated for cadmium, mercury and methylmercury (results not shown). Additional adjustment for “family size” and removing “BMIz” had no appreciable influence on our findings; findings without creatinine adjustment of urinary measures (results now shown) were similar to the adjusted estimates.

**Table 2:** Relative association between Asian identification, parental education, and their interaction on metal exposure, unadjusted and adjusted for potential confounders

Metal	Asian (ref = non-Asian)	Parental education	Parental education	Asian × parental education (< high school)	Asian × parental education (high school–some college)
		< High school (ref = college+)	High school-some college (ref = college+)		
	exp[β (95% CI)]	exp[β (95% CI)]	exp[β (95% CI)]	exp[β (95% CI)]	exp[β (95% CI)]
Without adjustment^a^					
Pb	1.46(1.20, 1.78)	1.26(1.10, 1.44)	1.20(1.12, 1.28)	1.04(0.79, 1.37)	0.79(0.62, 1.02)
Cd	1.35(1.24, 1.47)	1.04(0.97, 1.11)	0.99(0.94, 1.05)	1.43(1.10, 1.87)	1.12(0.96, 1.30)
Hg	1.71(1.46, 1.99)	0.95(0.85, 1.06)	0.92(0.83, 1.01)	1.63(1.08, 2.48)	1.35(1.07, 1.69)
MeHg	1.9(1.58, 2.29)	0.85(0.72, 1.00)	0.87(0.75, 1.00)	1.95(1.28, 2.97)	1.43(1.07, 1.90)
As	1.74(1.3, 2.33)	1.07(0.91, 1.24)	1.08(0.93, 1.25)	1.52(0.69, 3.34)	1.15(0.71, 1.86)
As(OH)3	1.10(0.9, 1.34)	1.08(0.92, 1.28)	0.95(0.84, 1.08)	1.11(0.66, 1.85)	0.96(0.69, 1.34)
DMA	1.55(1.29, 1.86)	1.04(0.93, 1.17)	1.01(0.92, 1.11)	1.31(0.83, 2.07)	1.09(0.77, 1.54)
MMA	1.27(1.03, 1.57)	0.93(0.82, 1.05)	0.96(0.85, 1.10)	1.23(0.80, 1.88)	0.86(0.66, 1.13)
With adjustment^b^					
Pb	1.34(1.16, 1.55)	1.19(1.01, 1.41)	1.17(1.07, 1.27)	1.16(0.87, 1.54)	0.84(0.64, 1.09)
Cd	1.32(1.20, 1.45)	1.01(0.94, 1.09)	0.98(0.92, 1.03)	1.40(1.07, 1.84)	1.03(0.89, 1.20)
Hg	1.64(1.36, 1.96)	0.90(0.80, 1.01)	0.90(0.81, 1.00)	1.46(0.95, 2.24)	1.24(0.98, 1.57)
MeHg	1.81(1.46, 2.24)	0.83(0.73, 0.95)	0.87(0.77, 0.98)	1.71(1.00, 2.92)	1.29(0.97, 1.72)
As	1.78(1.26, 2.50)	1.07(0.92, 1.25)	1.09(0.93, 1.28)	1.26(0.53, 2.98)	1.07(0.64, 1.80)
As(OH)3	1.10(0.85, 1.41)	0.94(0.75, 1.17)	0.93(0.80, 1.09)	1.08(0.58, 1.99)	0.86(0.54, 1.36)
DMA	1.58(1.28, 1.96)	1.03(0.91, 1.17)	1.04(0.93, 1.15)	1.07(0.66, 1.74)	0.98(0.66, 1.45)
MMA	1.28(0.99, 1.65)	0.93(0.81, 1.06)	1.02(0.91, 1.15)	1.00(0.63, 1.59)	0.74(0.53, 1.04)

‘non-Asian’ includes all non-Hispanic White, Black, Mexican, other Hispanic and other non-Hispanic participants

*CI* confidence interval, *Pb* lead, *Cd* cadmium, *Hg* mercury, *MeHg* methylmercury, *As* arsenic, *As(OH)3* arsenous acid, *MMA* monomethylarsonic acid, *DMA* dimethylarsinic acid

^a^Models are linear regression of log transformed metal concentrations (ln(µg/L); ln(µg/dL) for Pb) comparing Asian (N = 460) vs. non-Asian (N = 4833), adjusted for parental education, and an interaction term between Asian and parental education. Shown are exponentiated β-coefficients (95% CI), which can be interpreted as relative associations or ratios of geometric means of the metal concentrations

^b^Models further adjust for age, sex/gender, income, US-born, BMI-Z score, cycle, with sampling weighting applied; urinary measurements adjusted for creatinine

The average exposure to metals was mostly higher for foreign-born (vs. US-born) children/adolescents among Asian and non-Asian participants, except for arsenic which was elevated among both-foreign and US-born Asian participants compared to other groups (Fig. S4). In the adjusted race/ethnicity stratified models, higher lead exposure among foreign born individuals was suggested, however confidence intervals were wide possibly due to the limited sample size among subgroups (Table S2). There was no clear pattern for metal concentrations across family income levels suggested (Fig. S5), only lead levels were higher among individuals with the lowest family income level (< $20,000) compared to the highest level ($75,000+), among the Asian and non-Asian groups (Table S3).

### Mediation by Fish/Shellfish Consumption

Consumption of fish and shellfish in the past month, respectively, mediated 9.1% (95%CI 2.6%, 17.3%) and 5.7% (0.5%, 12.0%) of the estimated total difference in mercury exposure between Asian vs. non-Asian participants, and similarly, 8.9% (2.5%, 15.7%) and 5.3% (0.8%, 11.3%) in relation to methylmercury exposure (Table S4). For other metal exposures the ACME interval estimates did not indicate mediation related to fish or shellfish consumption.

## Discussion

Our study based on NHANES data (2015–2018) suggests that Asian children and adolescents in the U.S. have higher exposure to lead, cadmium, mercury/methylmercury, and arsenic than their non-Asian peers, even after adjusting for socio-demographic characteristics and BMIz. Notably, among Asian children and youth, lower parental educational attainment related to higher metal concentrations, in line with a 2011–2012 NHANES study indicating inverse associations between education and lead/cadmium among Asian participants ≥ 6 years [8]. Our results further suggest that the elevated mercury and methylmercury exposures of Asian children and teens, thus the disparity, can be partially explained by fish and shellfish consumption. Overall, our findings indicate that Asian children and youth may be disproportionally affected by toxic metal exposure in the U.S., and those from low parental educational backgrounds may be particularly at risk warranting further research to identify and consequently prevent exposure; including the consideration of fish and shellfish consumption, along with other unexamined dietary factors, as potential exposure sources especially for mercury and methylmercury, and possibly arsenic exposure, among U.S.-Asian children and adolescents.

Our findings align with studies of earlier NHANES cycles among adults and assessments among adults in the U.S., UK, and Canada [2, 3, 5, 8, 9, 11, 13, 15]. In addition to the blood/urine samples which capture more recent exposures, previous studies using hair biomarkers indicative of longer-term exposure also suggested sustainedly higher metal exposure among Asian population groups in the U.S. compared to other racial/ethnic groups [13, 28].

Metal concentrations in the U.S.-Asian population have been shown to be generally lower than in Asian countries [16–18]. In our study, U.S.-born children and adolescents had generally lower exposures than foreign-born peers, overall as well as within the Asian group. Notably, total urinary arsenic was similarly elevated among Asian children/adolescents born in the US as among those born abroad. An earlier NHANES 2011–2012 analysis (aged ≥ 6 years) reported lower blood cadmium and lead among U.S.-born Asian participants compared to other US-born racial/ethnic groups [8], which was not observed in our study. Moreover, SES based on income alone did not play a similar role as parental education in our analyses in contrast to a previous NHANES 2011–2012 study, which observed a negative association between income and blood cadmium in Asian participants;^35^ this difference might be due to our focus on children and adolescents, for whom parental education is a strong predictor of health outcomes. Future research should consider additional SES indicators such as neighborhood deprivation in relation to metal exposure in children, which was suggested to be associated with higher urinary lead levels among Asian girls in Northern California [20]. Also, arsenic exposure among disproportionally affected Asian subgroups in the US should be further examined to identify and mitigate potential exposure sources.

Our model-based mediation analysis suggested that recent fish/shellfish consumption partially mediated the elevated mercury and methylmercury concentrations among Asian participants. Asian participants also consumed fish/shellfish more frequently, consistent with previous NHANES studies [9, 10]. Considering that heavy metal exposure in general is only in part attributable to specific dietary factors [9, 25, 26], the consumption of fish/shellfish alone likely accounts for a considerable proportion of the racial/ethnic differences in certain metal exposure [27], with possibly other dietary and non-dietary factors also playing a role [27], We could not assess species-specific fish and shellfish products due to substantial missingness in the data, and data on other dietary factors (e.g., rice) were unavailable in these NHANES cycles. To better understand and mitigate heavy metal exposure, especially among children from diverse backgrounds, further research is needed. For example, detailed dietary intake assessments among children could help to identify specific food items contributing to metal exposure. Additionally, investigating contamination levels in fish products and other foodstuffs will inform evidence-based nutrition guidelines that should ensure safety levels for children and pregnant persons; thus contributing to reductions of harmful heavy metal exposure.

There were several limitations in the present study. Since NHANES is a cross-sectional assessment, the metal concentration measurements reflect short-term rather than long-term exposures, as metals have relatively short biological half-lives between days such as urinary arsenic and months, such as blood cadmium. Since the 2011–2012 cycle, NHANES has oversampled Asian participants to ensure reliable national estimates for this subgroup, potentially affecting our analytic sample’s representativeness [33]. However, with the application of survey weight adjustment, potential bias due to such complex survey design and unequal probabilities of selection was less of a concern [37, 38]. We used combined non-Asian participants as the primary reference group for comparison with Asian participants in order to retain a sufficient sample size. Non-Hispanic White participants were used in sensitivity analysis as reference group to address the influence of potential biases related to the heterogeneity within the non-Asian group; the distributions of SES factors were also more comparable between Asian and White participants than with other subgroups. As the largest racial/ethnic group in the US, the non-Hispanic Whites had been the most common reference group for assessing racial/ethnic health disparities in previous studies, with which our results could be more directly comparable; however, the estimates using either non-Asian or White participants as the comparison reference were overall consistent. We did not break down ethnic background within the Asian group (such as Vietnamese, Chinese, Japanese, etc.) due to the sample size limitations; accounting for different Asian subgroups by origin in the U.S. should be a next step to illustrate environmental inequities Asian children and youth are facing, and to better understand the impact of seafood consumption and other dietary factors for a diverse U.S. population with various dietary habits. In addition to seafood, certain vegetables and cereal grains such as rice could be another important source of arsenic exposure for Asians as suggested by previous studies [24]. However, data on rice consumption were not available for the 2015–2018 NHANES cycles.

### New Contribution to the Literature

Based on the representative NHANES data, our findings newly suggest a disparity such that U.S.-Asian children and adolescents are exposed to higher levels of several heavy metals than children and adolescents from other racial/ethnic groups in the U.S., with those with lower parental education being at particular risk for elevated exposure. Dietary habit related intake of fish/shellfish and possibly other food stuff should be considered as potentially preventable exposure source. Finally, our findings highlight inequities regarding harmful metal exposure experienced by a growing Asian young population in the U.S., suggesting a susceptible target group for prevention.

## Supplementary Information

Below is the link to the electronic supplementary material.

Supplementary file1 (PDF 993 KB)

## Data Availability

The datasets generated during and/or analyzed during the current study are available from the corresponding author on reasonable request.

## References

[1] Rehman K, Fatima F, Waheed I, Akash MSH. Prevalence of exposure of heavy metals and their impact on health consequences. J Cell Biochem. 2018;119:157–84. 10.1002/jcb.26234. 28643849 10.1002/jcb.26234

[2] Hightower JM, O’Hare A, Hernandez GT. Blood mercury reporting in NHANES: identifying Asian, Pacific Islander, Native American, and multiracial groups. Environ Health Perspect. 2006;114:173–5. 16451850 10.1289/ehp.8464 PMC1367827

[3] Shim YK, Lewin MD, Ruiz P, Eichner JE, Mumtaz MM. Prevalence and associated demographic characteristics of exposure to multiple metals and their species in human populations: the United States NHANES, 2007–2012. J Toxicol Environ Health A. 2017;80:502. 28703686 10.1080/15287394.2017.1330581 PMC5693367

[4] Tyrrell J, Melzer D, Henley W, Galloway TS, Osborne NJ. Associations between socioeconomic status and environmental toxicant concentrations in adults in the USA: NHANES 2001–2010. Environ Int. 2013;59:328–35. 23892225 10.1016/j.envint.2013.06.017

[5] Mortensen ME, Caudill SP, Caldwell KL, Ward CD, Jones RL. Total and methyl mercury in whole blood measured for the first time in the U.S. population: NHANES 2011–2012. Environ Res. 2014;134:257. 25173092 10.1016/j.envres.2014.07.019 PMC5584810

[6] Sanders AP, Mazzella MJ, Malin AJ, Hair G, Busgang SA, Saland JM, et al. Combined exposure to lead, cadmium, mercury, and arsenic and kidney health in adolescents age 12–19 in NHANES 2009–2014. Environ Int. 2019;131: 104993. 31326826 10.1016/j.envint.2019.104993 PMC6750805

[7] National Report on Human Exposure to Environmental Chemicals | CDC [cited 2022 Apr 19]. Available from: https://www.cdc.gov/exposurereport/.

[8] Awata H, Linder S, Mitchell LE, Delclos GL. Biomarker levels of toxic metals among Asian populations in the United States: NHANES 2011–2012. Environ Health Perspect. 2017;125:306. 27517362 10.1289/EHP27 PMC5332180

[9] Awata H, Linder S, Mitchell LE, Delclos GL. Association of dietary intake and biomarker levels of arsenic, cadmium, lead, and mercury among asian populations in the United States: NHANES 2011–2012. Environ Health Perspect. 2017;125:314–23. 10.1289/EHP28. 27586241 10.1289/EHP28 PMC5332183

[10] Liu Y, Buchanan S, Anderson HA, Xiao Z, Persky V, Turyk ME. Association of methylmercury intake from seafood consumption and blood mercury level among the Asian and Non-Asian populations in the United States. Environ Res. 2018;160:212–22. 29020643 10.1016/j.envres.2017.09.031

[11] Yeter D, Portman MA, Aschner M, Farina M, Chan WC, Hsieh KS, et al. Ethnic Kawasaki disease risk associated with blood mercury and cadmium in U.S. children. Int J Environ Res Public Health. 2016;13:101. 26742052 10.3390/ijerph13010101 PMC4730492

[12] Wang X, Mukherjee B, Batterman S, Harlow SD, Park SK. Urinary metals and metal mixtures in midlife women: the Study of Women’s Health Across the Nation (SWAN). Int J Hyg Environ Health. 2019;222:778. 31103473 10.1016/j.ijheh.2019.05.002 PMC6583796

[13] Brima EI, Haris PI, Jenkins RO, Polya DA, Gault AG, Harrington CF. Understanding arsenic metabolism through a comparative study of arsenic levels in the urine, hair and fingernails of healthy volunteers from three unexposed ethnic groups in the United Kingdom. Toxicol Appl Pharmacol. 2006;216:122–30. 16762385 10.1016/j.taap.2006.04.004

[14] Lye E, Legrand M, Clarke J, Probert A. Blood total mercury concentrations in the Canadian population: Canadian health measures survey cycle 1, 2007–2009. Can J Public Health. 2013;104: e246. 23823890 10.17269/cjph.104.3772 PMC6974123

[15] Dix-Cooper L, Kosatsky T. Blood mercury, lead and cadmium levels and determinants of exposure among newcomer South and East Asian women of reproductive age living in Vancouver, Canada. Sci Total Environ. 2018;619–620:1409–19. 29734617 10.1016/j.scitotenv.2017.11.126

[16] Choi W, Kim S, Baek YW, Choi K, Lee K, Kim S, et al. Exposure to environmental chemicals among Korean adults-updates from the second Korean National Environmental Health Survey (2012–2014). Int J Hyg Environ Health. 2017;220:29–35. 27816434 10.1016/j.ijheh.2016.10.002

[17] Nakayama SF, Iwai-Shimada M, Oguri T, Isobe T, Takeuchi A, Kobayashi Y, et al. Blood mercury, lead, cadmium, manganese and selenium levels in pregnant women and their determinants: the Japan Environment and Children’s Study (JECS). J Expo Sci Environ Epidemiol. 2019;29(5):633–47. 31000792 10.1038/s41370-019-0139-0 PMC6760604

[18] Barnett-Itzhaki Z, Esteban López M, Puttaswamy N, Berman T. A review of human biomonitoring in selected Southeast Asian countries. Environ Int. 2018;116:156–64. 29684824 10.1016/j.envint.2018.03.046

[19] Montazeri P, Thomsen C, Casas M, de Bont J, Haug LS, Maitre L, et al. Socioeconomic position and exposure to multiple environmental chemical contaminants in six European mother-child cohorts. Int J Hyg Environ Health. 2019;222:864. 31010791 10.1016/j.ijheh.2019.04.002 PMC8713641

[20] Gonzales FA, Jones RR, Deardorff J, Windham GC, Hiatt RA, Kushi LH. Neighborhood deprivation, race/ethnicity, and urinary metal concentrations among young girls in California. Environ Int. 2016;91:29. 26908165 10.1016/j.envint.2016.02.004 PMC6360017

[21] Kaplowitz SA, Perlstadt H, Dziura JD, Post LA. Behavioral and environmental explanations of elevated blood lead levels in immigrant children and children of immigrants. J Immigr Minor Health. 2016;18:979–86. 26163335 10.1007/s10903-015-0243-8

[22] Rashid A, Bhat RA, Qadri H, Mehmood MA, Shafiq-ur-Rehman. Environmental and socioeconomic factors induced blood lead in children: an investigation from Kashmir. India Environ Monit Assess. 2019;191:1–10. 10.1007/s10661-019-7220-y. 10.1007/s10661-019-7220-y 30648205

[23] Kowalska M, Kulka E, Jarosz W, Kowalski M. The determinants of lead and cadmium blood levels for preschool children from industrially contaminated sites in Poland. Int J Occup Med Environ Health. 2017;31:351–9. 29072712 10.13075/ijomeh.1896.01153

[24] Davis MA, Mackenzie TA, Cottingham KL, Gilbert-Diamond D, Punshon T, Karagas MR. Rice consumption and urinary arsenic concentrations in U.S. children. Environ Health Perspect. 2012;120:1418–24. 10.1289/ehp.1205014. 23008276 10.1289/ehp.1205014 PMC3491944

[25] Jain RB. Contribution of diet and other factors for urinary concentrations of total arsenic and arsenic species: data for US children, adolescents, and adults. Environ Sci Pollut Res. 2021;28:50094–116. 10.1007/s11356-021-14230-9. 10.1007/s11356-021-14230-9 33948846

[26] Mahaffey KR, Clickner RP, Bodurow CC. Blood organic mercury and dietary mercury intake: National Health and Nutrition Examination Survey, 1999 and 2000. Environ Health Perspect. 2004;112:562–70. 15064162 10.1289/ehp.6587 PMC1241922

[27] Tsuchiya A, Hinners TA, Krogstad F, White JW, Burbacher TM, Faustman EM, et al. Longitudinal mercury monitoring within the Japanese and Korean communities (United States): implications for exposure determination and public health protection. Environ Health Perspect. 2009;117:1760. 20049129 10.1289/ehp.0900801 PMC2801193

[28] Buchanan S, Targos L, Nagy KL, Kearney KE, Turyk M. Fish consumption and hair mercury among Asians in Chicago. J Occup Environ Med. 2015;57:1325–30. 26641830 10.1097/JOM.0000000000000560

[29] Phelan JC, Link BG, Tehranifar P. Social conditions as fundamental causes of health inequalities: theory, evidence, and policy implications. J Health Soc Behav. 2010;51(Suppl):S28-40. 20943581 10.1177/0022146510383498

[30] VanderWeele TJ, Robinson WR. On causal interpretation of race in regressions adjusting for confounding and mediating variables. Epidemiology. 2014;25:473. 24887159 10.1097/EDE.0000000000000105 PMC4125322

[31] Edgoose JYC, Carvajal DN, Reavis KMP, Yogendran L, Echiverri AT, Rodriguez JE. Addressing and dismantling the legacy of race and racism in academic medicine: a socioecological framework. J Am Board Fam Med. 2022;35:1239–45. 36396417 10.3122/jabfm.2022.220050R2 PMC9983036

[32] Center for Health Statistics N. Vital and health statistics series 2, number 184 April 2020: sample design and estimation procedures data evaluation and methods research. 2015 [cited 2021 Sept 15]; Available from: https://www.cdc.gov/nchs/products/index.htm.

[33] NHANES Tutorials - Module 2 - Sample Design [cited 2022 Sept 21]. Available from: https://wwwn.cdc.gov/nchs/nhanes/tutorials/Module2.aspx.

[34] NHANES Questionnaires, Datasets, and Related Documentation [cited 2023 July 22]. Available from: https://wwwn.cdc.gov/nchs/nhanes/default.aspx.

[35] Yeter D, Banks EC, Aschner M. Disparity in risk factor severity for early childhood blood lead among predominantly African-American Black Children: the 1999 to 2010 US NHANES. Int J Environ Res Public Health. 2020;17:1552. 32121216 10.3390/ijerph17051552 PMC7084658

[36] Tingley D, Yamamoto T, Hirose K, Keele L, Imai K. mediation: R package for causal mediation analysis. J Stat Softw. 2014;59:1–38.26917999

[37] Paulose-Ram R, Burt V, Broitman L, Ahluwalia N. Overview of Asian American data collection, release, and analysis: National Health and Nutrition Examination Survey 2011–2018. Am J Public Health. 2017;107:916. 28426300 10.2105/AJPH.2017.303815 PMC5425904

[38] Center for Health Statistics N. Vital and health statistics series 2, number 184 April 2020: sample design and estimation procedures data evaluation and methods research. 2015 [cited 2022 Sept 26]. Available from: https://www.cdc.gov/nchs/products/index.htm.

