# Supplementary material for: Exposure to Lead, Cadmium, Mercury and Arsenic Among Asian and Non-Asian Children and Adolescents in the United States: NHANES 2015–2018
Source: J Immigr Minor Health. 2025 Jan 3;27(2):258–67. doi: 10.1007/s10903-024-01634-1 (PMC11903527; doi:10.1007/s10903-024-01634-1)
Supplement: Supplementary file 1 — Supplementary file1 (PDF 993 KB) [file 10903_2024_1634_MOESM1_ESM.pdf]

Article title: Exposure to Lead, Cadmium, Mercury and Arsenic Among Asian and Non-Asian Children and Adolescents in the United States: NHANES 2015-2018

Journal name: Journal of Immigrant and Minority Health

Authors:

Lanxin Song[1] and Ondine S. von Ehrenstein[1,2]

[1] Department of Epidemiology, Fielding School of Public Health, University of California, Los Angeles, CA, United States
[2] Department of Community Health Sciences, Fielding School of Public Health, University of California, Los Angeles, CA, United States

Address correspondence to:

Lanxin Song, University of California, Los Angeles, PO Box 951772, Los Angeles, CA 90095-1772, ORCID: 0000-0002-7869-2700, lsong07@g.ucla.edu

Dr. Ondine von Ehrenstein, University of California, Los Angeles, PO Box 951772, Los Angeles, CA 90095-1772, ovehren@ucla.edu

Description: This supplementary material contains supplementary tables and figures in the order referenced in the text.

Table S1. Differences in log-transformed metal concentrations comparing parental education levels in Asian and non-Asian subgroups. [a]

	Asian		non-Asian	
	<High School	High School-Some College	<High School	High School-Some College
Pb	0.25 (0.00, 0.50)	-0.09 (-0.30, 0.12)	0.18 (0.01, 0.35)	0.16 (0.07, 0.24)
Cd	0.37 (0.04, 0.70)	-0.01 (-0.17, 0.16)	0.01 (-0.06, 0.08)	-0.03 (-0.09, 0.03)
Hg	0.26 (-0.19, 0.71)	0.05 (-0.25, 0.36)	-0.10 (-0.22, 0.01)	-0.10 (-0.21, 0.00)
MeHg	0.37 (-0.15, 0.88)	0.08 (-0.27, 0.44)	-0.18 (-0.31, -0.05)	-0.14 (-0.26, -0.01)
As	0.29 (-0.86, 1.44)	0.39 (-0.08, 0.85)	0.04 (-0.13, 0.2)	0.03 (-0.12, 0.18)
As(OH)3	0.03 (-0.61, 0.67)	-0.08 (-0.52, 0.36)	-0.08 (-0.29, 0.12)	-0.12 (-0.27, 0.04)
DMA	0.10 (-0.57, 0.77)	0.21 (-0.08, 0.49)	0.01 (-0.10, 0.13)	0.00 (-0.10, 0.09)
MMA	-0.15 (-0.51, 0.22)	-0.2 (-0.50, 0.11)	-0.08 (-0.21, 0.05)	-0.02 (-0.12, 0.08)

[a] Models are linear regression of log transformed metal concentrations (ln(μg/L; ln(μg/dL) for Pb), adjusting for age, gender, income, US-born, BMI-Z and cycle, with sampling weighting applied; urinary measurements adjusted for creatinine; reference ="College+". Shown are β-coefficients (95% CI).

Table S2. Differences in log-transformed metal concentrations comparing foreign-born vs. US-born among Asian and non-Asian Participants. [a]

	Asian	non-Asian
	Foreign-born	Foreign-born
Pb	0.31(0.21, 0.41)	0.14 (0.01, 0.28)
Cd	0.14(-0.03, 0.32)	0.01 (-0.08, 0.09)
Hg	-0.11(-0.52, 0.31)	0.15 (-0.02, 0.32)
MeHg	-0.07(-0.54, 0.4)	0.27 (0.06, 0.49)
As	-0.04 (-0.57, 0.49)	0.3 (0.10, 0.49)
As(OH)3	0.12 (-0.48, 0.72)	0.41 (0.13, 0.7)
DMA	0.10 (-0.35, 0.55)	0.30 (0.1, 0.51)
MMA	0.18 (-0.21, 0.57)	0.33 (0.1, 0.55)

[a] Models are linear regression of log transformed metal concentrations (ln(μg/L; ln(μg/dL) for Pb), adjusting for age, gender, income, parental education, BMI-Z and cycle, with sampling weighting applied; urinary measurements adjusted for creatinine; reference=”US-born”. Shown are β-coefficients (95% CI).

Table S3. Differences in log-transformed metal concentrations comparing family income levels in Asian and non-Asian subgroups. [a]

Subgroup	Metal	Family income level		
		<$20,000	$20,000-$45,000	$45000-$75,000
Asian	Pb	0.46 (0.08, 0.84)	0.11(-0.07, 0.30)	-0.07 (-0.27, 0.12)
	Cd	0.20 (-0.06, 0.47)	-0.06 (-0.3, 0.17)	-0.15 (-0.33, 0.02)
	Hg	0.11(-0.53, 0.76)	0.03 (-0.44, 0.5)	0.14 (-0.31, 0.58)
	MeHg	-0.04 (-0.73, 0.66)	0.01 (-0.51, 0.52)	0.07 (-0.47, 0.6)
	As	-0.38 (-1.65, 0.89)	-0.31 (-0.85, 0.23)	0.12 (-0.46, 0.69)
	As(OH)3	-0.36 (-0.96, 0.24)	0.00 (-0.47, 0.47)	0.11 (-0.24, 0.45)
	DMA	-0.34 (-1.03, 0.35)	-0.13 (-0.52, 0.27)	-0.11 (-0.49, 0.27)
	MMA	-0.31 (-0.66, 0.05)	0.16 (-0.14, 0.46)	0.04 (-0.34, 0.43)
non-Asian	Pb	0.19 (0.07, 0.30)	0.06 (-0.04, 0.17)	0.07 (-0.04, 0.17)
	Cd	0.04 (-0.02, 0.10)	0.02 (-0.05, 0.08)	0.02 (-0.05, 0.10)
	Hg	0.07 (-0.04, 0.18)	0.03 (-0.06, 0.12)	-0.04 (-0.15, 0.06)
	MeHg	0.02 (-0.08, 0.13)	0.00 (-0.11, 0.11)	-0.10 (-0.21, 0.00)
	As	-0.06 (-0.19, 0.06)	-0.13 (-0.24, -0.01)	-0.04 (-0.20, 0.12)
	As(OH)3	0.06 (-0.17, 0.29)	0.03 (-0.13, 0.18)	0.02 (-0.16, 0.19)
	DMA	-0.06 (-0.17, 0.06)	-0.11 (-0.2, -0.01)	-0.07 (-0.19, 0.04)
	MMA	-0.10 (-0.24, 0.05)	-0.14 (-0.27, -0.02)	-0.10 (-0.23, 0.04)

[a] Models are linear regression of log transformed metal concentrations (ln(μg/L; ln(μg/dL) for Pb), adjusting for age, gender, US-born, parental education, BMI-Z and cycle, with sampling weighting applied; urinary measurements adjusted for creatinine; reference income=”$75,000+". Shown are β-coefficients (95% CI).

Table S4. Estimated Average Causal Mediation Effect (ACME), Proportion Mediated (PM), and Total Effects (TE) with 95% Confidence Intervals of Fish and Shellfish Consumption, Among Asian vs. non-Asian, Respectively.

		Fish	Shellfish
Metal	Estimator	Asians (n=358) vs. non-Asians (n=4197)	Asians (n=359) vs. non-Asians (n=4208)
Pb	ACME	-0.001 (-0.008, 0.006)	-0.002 (-0.008, 0.003)
	PM	-0.002 (-0.033, 0.025)	-0.006 (-0.034, 0.011)
	TE	0.248 (0.157, 0.333)	0.251 (0.167, 0.342)
Cd	ACME	0.000 (-0.007, 0.005)	-0.001 (-0.005, 0.004)
	PM	-0.001 (-0.023, 0.018)	-0.002 (-0.020, 0.011)
	TE	0.302 (0.209, 0.391)	0.306 (0.215, 0.398)
Hg	ACME	0.055 (0.015, 0.094)	0.033 (0.003, 0.065)
	PM	0.091 (0.026, 0.173)	0.057 (0.005, 0.120)
	TE	0.584 (0.412, 0.737)	0.581 (0.420, 0.742)
MeHg	ACME	0.064 (0.016, 0.115)	0.039 (0.006, 0.079)
	PM	0.090 (0.025, 0.157)	0.053 (0.008, 0.113)
	TE	0.713 (0.538, 0.887)	0.709 (0.534, 0.882)
As	ACME	0.056 (-0.004, 0.122)	0.042 (-0.013, 0.108)
	PM	0.093 (-0.007, 0.199)	0.063 (-0.024, 0.178)
	TE	0.592 (0.372, 0.814)	0.622 (0.412, 0.829)
As(OH)3	ACME	-0.003 (-0.018, 0.011)	-0.001 (-0.015, 0.012)
	PM	-0.015 (-0.309, 0.201)	-0.004 (-0.297, 0.312)
	TE	0.127 (-0.022, 0.281)	0.118 (-0.036, 0.276)
DMA	ACME	0.017 (-0.002, 0.04)	0.017 (-0.003, 0.043)
	PM	0.033 (-0.005, 0.084)	0.035 (-0.007, 0.092)
	TE	0.481 (0.358, 0.611)	0.486 (0.37, 0.604)
MMA	ACME	0.004 (-0.006, 0.019)	-0.002 (-0.017, 0.009)
	PM	0.013 (-0.022, 0.158)	-0.006 (-0.096, 0.05)
	TE	0.207 (0.077, 0.335)	0.202 (0.076, 0.333)

Note. 'non-Asian' includes all non-Hispanic White, Black, Mexican, other Hispanic and other non-Hispanic participants. Mediation models of metal measurements adjusted for age, sex/gender, income, US-born, BMI-Z score, cycle, parental education level, with sampling weighting applied; urinary measurements further adjusted for creatinine.

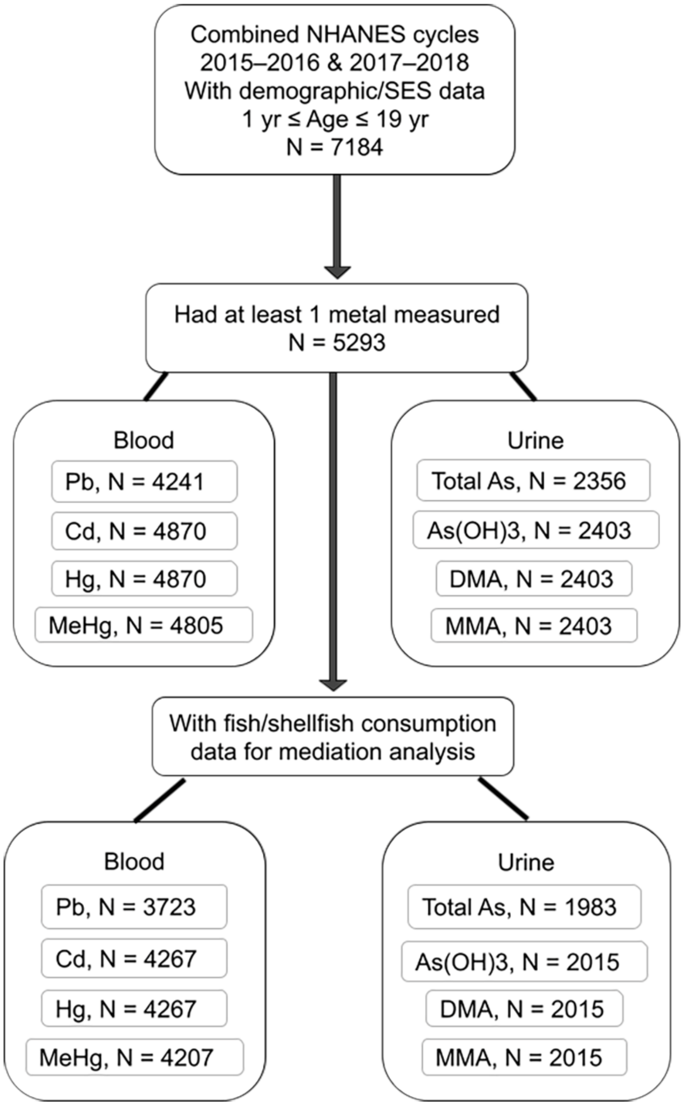

Figure S1. Flowchart derivation of study sample.
Note. NHANES = National Health and Nutrition Examination Survey. SES = socioeconomic status. Pb = lead. Cd = cadmium. Hg = mercury. MeHg = methylmercury. As = arsenic. As(OH)3 = arsenous acid. MMA = monomethylarsonic acid. DMA = dimethylarsinic acid.

Supplementary information for the study population: We calculated BMI z-scores (BMIz) for children and teens using the CDC 2000 growth charts (aged 2-19 years) and WHO growth standards (aged 12-24 months) by the LMS method [1–3]. Blood collection eligibility varied: in 2015-2016, ages 1-11 and half of those 12+ were eligible; in 2017-2018, ages 6+ were eligible for lead, while ages 1+ were eligible for other metals. For urine, ages 3-5 and a third of ages 6+ were eligible in both cycles. About 85% of this group had data on fish (n= 4555) and shellfish (n=4567) consumption in the month prior to the MEC examination, used for mediation analysis (Figure S1).

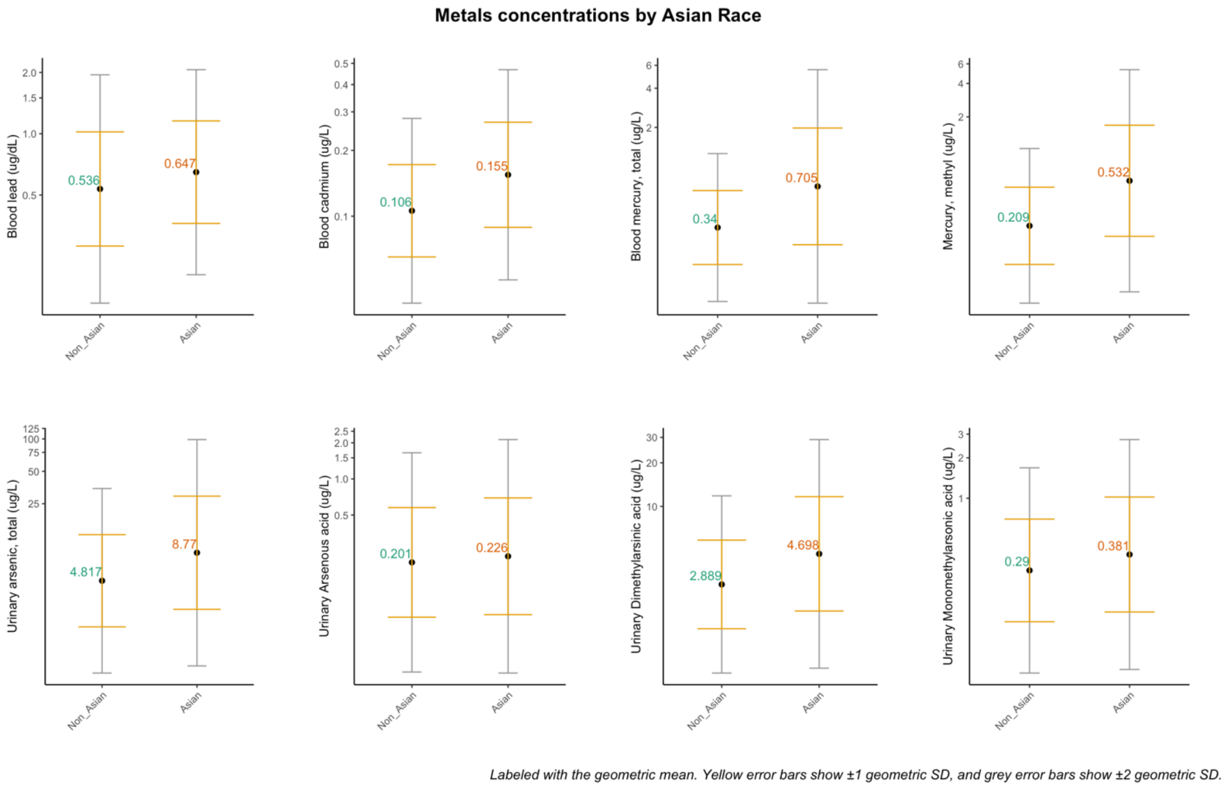

Figure S2. Distribution of metal concentrations for non-Asian and Asian participants aged 1-19 years in NHANES 2015-2018.

Note. NHANES = National Health and Nutrition Examination Survey. Figures are labeled with geometric means and error bars (±1 and ±2 geometric standard deviations).

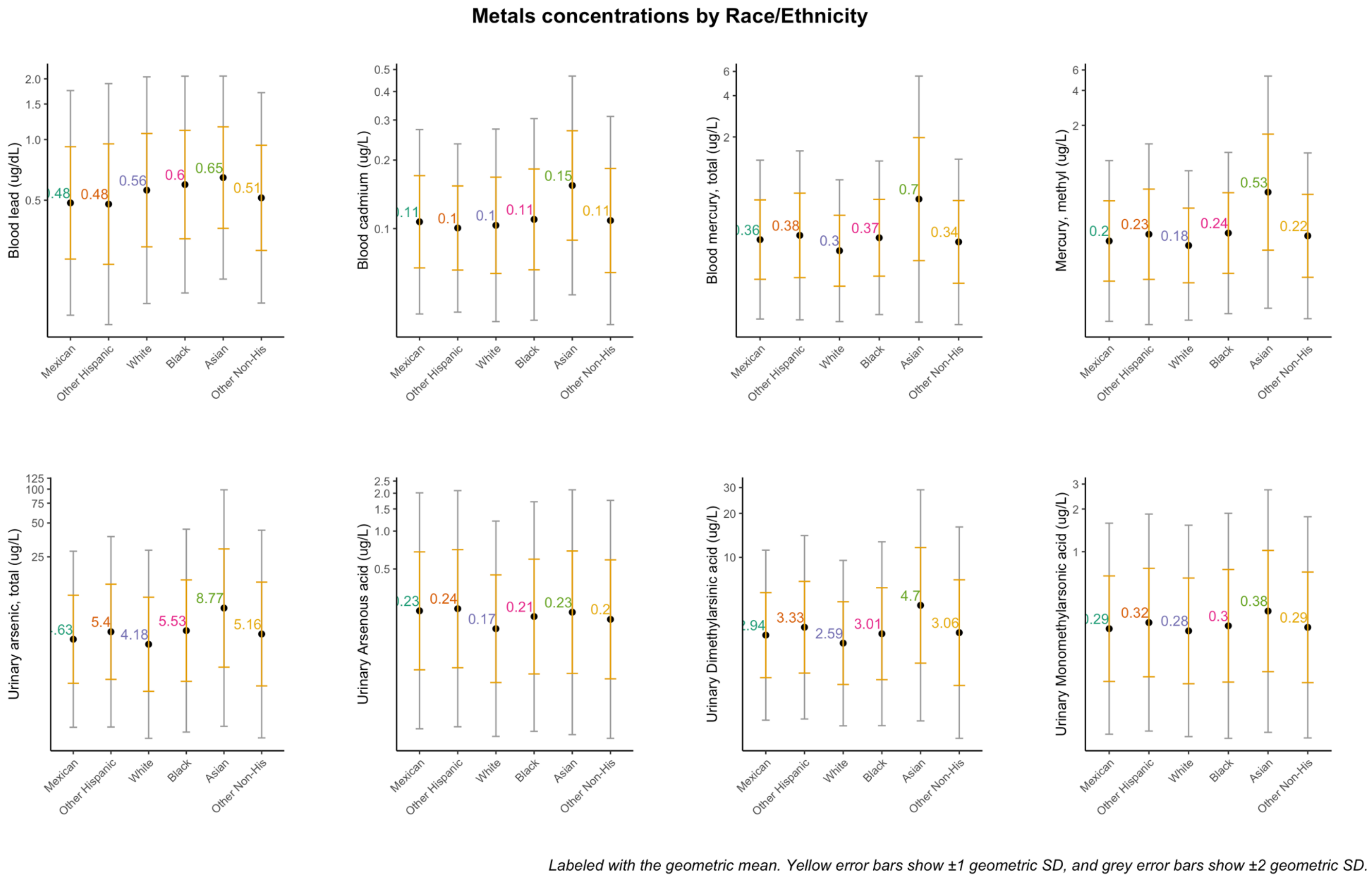

Figure S3. Boxplots of metal concentrations by race/ethnicity for participants aged 1-19 years in NHANES 2015-2018.

Note. NHANES = National Health and Nutrition Examination Survey. Figures are labeled with geometric means and error bars (±1 and ±2 geometric standard deviations).

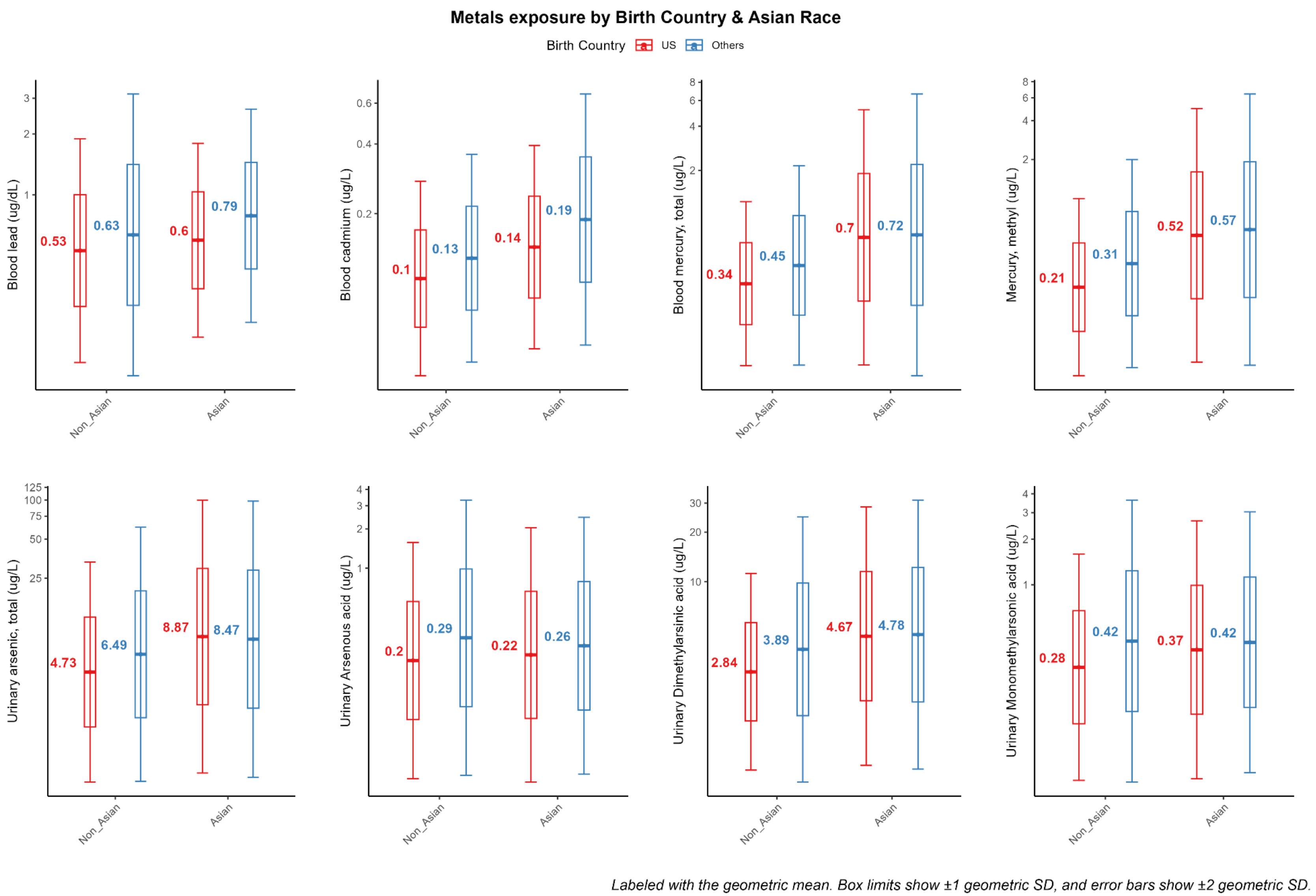

Figure S4. Boxplots of metal concentrations by country of birth for Asians and non-Asians aged 1-19 years in NHANES 2015-2018.

Note. NHANES = National Health and Nutrition Examination Survey. Figures are labeled with geometric means and error bars (±1 and ±2 geometric standard deviations).

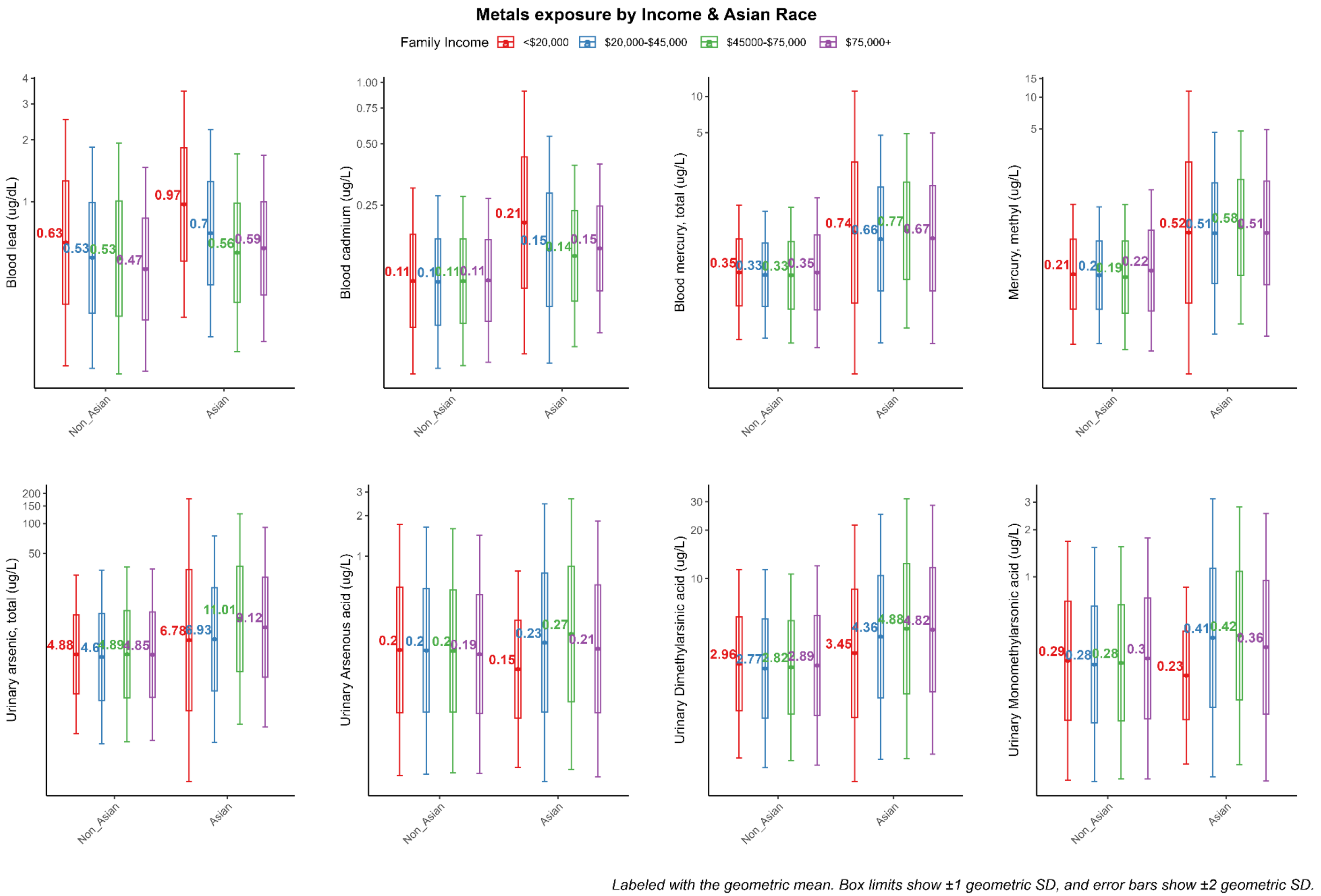

Figure S5. Boxplots of metal concentrations by family income levels for Asians and non-Asians aged 1-19 years in NHANES 2015-2018.

Note. NHANES = National Health and Nutrition Examination Survey. Figures are labeled with geometric means and error bars (±1 and ±2 geometric standard deviations).

References

1. Modified z-scores in the CDC growth charts [Internet]. 2016 [cited 2022 Dec 3]. Available from: https://www.cdc.gov/nccdphp/dnpa/growthcharts/resources/BIV-cutoffs.pdf
2. Sharma AK, Metzger DL, Daymont C, Hadjiyannakis S, Rodd CJ. LMS tables for waist-circumference and waist-height ratio Z-scores in children aged 5-19 y in NHANES III: association with cardio-metabolic risks. Pediatr Res [Internet]. 2015 [cited 2022 Mar 28];78:723–9. Available from: https://pubmed.ncbi.nlm.nih.gov/26331767/
3. Roundup of growth chart packages | Monica Gerber [Internet]. [cited 2022 Mar 28]. Available from: https://www.monicagerber.com/post/roundup-of-growth-chart-packages/
